# Seroprevalence, Genetic Characteristics, and Pathogenicity of Korean Porcine Sapeloviruses

**DOI:** 10.3390/v17070870

**Published:** 2025-06-20

**Authors:** Song-Yi Kim, Choi-Kyu Park, Gyu-Nam Park, SeEun Choe, Min-Kyung Jang, Young-Hyeon Lee, Yun Sang Cho, Dong-Jun An

**Affiliations:** 1 Virus Disease Division, Animal and Plant Quarantine Agency, Gimcheon 39660, Republic of Korea; songkim@korea.kr (S.-Y.K.); changep0418@korea.kr (G.-N.P.); ivvi59@korea.kr (S.C.); mkjang0506@korea.kr (M.-K.J.); yhlee916@korea.kr (Y.-H.L.); choys@korea.kr (Y.S.C.); 2 College of Veterinary Medicine & Institute for Veterinary Biomedical Science, Kyungpook National University, Daegu 41566, Republic of Korea

**Keywords:** PSV, seroprevalence, phylogenetic tree, pig, clinical signs

## Abstract

Although porcine sapelovirus (PSV) is generally subclinical, it can cause a wide range of clinical signs in some individuals, including respiratory distress, acute diarrhea, pneumonia, skin lesions, reproductive failure, and neurological diseases. In this study, we investigated the prevalence and genotype of PSV isolated from domestic pigs and wild boars in Korea. We also analyzed potential recombination events, and assessed the pathogenicity of the virus through animal experiments. In wild boars, the prevalence of PSV antibodies decreased slightly (by 1.8%) over 5 years (from 2019 to 2024); however, prevalence increased significantly (by 17.8%) in breeding sows. In samples from animals with diarrhea and respiratory clinical signs, the prevalence of PSV alone was 21.1%, whereas the prevalence of PSV mixed with other pathogens was also 21.1%. The whole genome of the PSV/Goryeong/KR-2019 strain isolated from a piglet with diarrhea was closely related to the Jpsv447 strain isolated in Japan in 2009, and recombination analysis predicted that the PSV/Goryeong/KR-2019 strain was generated by genetic recombination between the KS05151 strain and the Jpsv447 strain. However, when the PSV/Goryeong/KR-2019 strain was orally administered to 5-day-old suckling pigs, diarrhea clinical signs were mild, and no significant changes were observed in villus height and ridge depth in the duodenum, jejunum, or ileum. In addition, no neurological clinical signs were observed when the isolated virus was administered to 130-day-old pigs, and no specific lesions were found upon histopathological examination of brain tissue. In conclusion, PSV/Goryeong/KR-2019 appears to be a weakly pathogenic virus that does not cause severe diarrhea in suckling pigs, and does not cause neurological clinical signs in fattening pigs. Therefore, it is presumed that most PSVs detected in Korean pig farms are weakly pathogenic strains.

## 1. Introduction

Previously, porcine sapelovirus (PSV) was classified as a porcine enterovirus, along with porcine teschovirus (PTV) and porcine enterovirus (PEV); however, it was later reclassified as a separate genus based on genetic sequence analysis [1,2,3]. Taxonomically, PSV is currently classified as *Sapelovirus A*, which, together with *Sapelovirus B*, belongs to the genus *Sapelovirus* within the family *Picornaviridae* [3,4,5,6]. The PSV genome is a positive-sense, single-stranded RNA of approximately 7.5–8.3 kb in length, sharing the typical genomic organization of picornaviruses: a 5′ untranslated region (UTR), a single large open reading frame (ORF), a 3′ UTR, and a poly (A) tail [7,8]. The ORF encodes a polyprotein precursor of approximately 2331 amino acids, which is cleaved by viral proteases to yield 12 mature proteins: a leader protein (L), four structural proteins (VP1–VP4) that form the viral capsid, and seven nonstructural proteins (2A, 2B, 2C, 3A, 3B, 3C and 3D) involved in viral replication and processing [9,10]. Among them, VP1, the most variable and immunodominant protein, plays a crucial role in determining genetic relationships among picornaviruses [11,12]. PSV consists of only one serotype [13], but phylogenetic analysis has divided the PSV genome into genotypes 1 and 2 [14,15,16,17,18].

PSV, an important pathogen that primarily infects domestic pigs and wild boars, is transmitted mainly through the fecal–oral route [10,13,19,20]. While PSV is often subclinical, it can also cause a wide range of clinical manifestations such as respiratory distress, acute diarrhea, pneumonia, skin lesions, reproductive failure, and severe neurological disorders such as polioencephalomyelitis [10,11,13,21,22]. In particular, PSV co-infects hosts along with other enteric pathogens; these infections can occur in symptomatic and subclinical pigs, making detection, control, and prevention of disease more complicated [8,10,23,24,25,26,27]. Severe cases can result in high morbidity and mortality, leading to significant economic losses to the swine industry.

PSV infection was first identified in the United Kingdom in 1958 [28], and has since been reported in various countries, including Canada, Japan, Australia, Brazil, Spain, South Korea, Italy, and China [8,9,10,11,13,25,27,29,30]. Studies of PSV report antigen prevalences ranging from 7.1% in India [29] to 71.0% in Hungary [31]. The virus has also been detected in wild boars, with a prevalence of 6.4% in Spain [26] and 27.8% in the Czech Republic [27]. In Zambia, PSV was identified in 94% of fattening pigs and in 36.2% of suckling pigs, indicating widespread circulation in swine populations [23]. Additionally, serological studies in China report a high antibody prevalence (79.3%), while antigen detection in fecal samples reached 44%, further supporting the significant presence of the virus in commercial pig farms [16]. Although several studies have reported PSV infections in South Korea, there is limited knowledge regarding nationwide molecular and serological prevalence, and the pathogenicity of prevalent PSV strains in pigs has not been investigated.

Therefore, this study aimed to investigate changes in the prevalence of PSV antibodies in domestic pigs and wild boars in South Korea over the past 5 years, and to analyze the genotypes present in domestic pigs. In addition, we investigated clinical signs such as diarrhea and neurological clinical signs after re-inoculation of pigs with isolated PSV, and used the results to infer the pathogenicity of PSV prevalent in South Korea.

## 2. Materials and Methods

### 2.1. Sample Collection

To detect PSV, pigs (raised on pig farms nationwide) with diarrhea and respiratory clinical signs were necropsied, and various tissue samples were obtained. Fifty-seven samples (one sample comprised of a mixture of feces, intestinal tissue, and lung tissue from a pig) were collected from 20 farms and sent to the laboratory for routine diagnostic analysis. For the sero-epidemiological survey, the number of samples obtained from sows and wild boars was determined using the “Sample size to estimate a proportion” tool in the EpiTools program (https://epitools.ausvet.com.au/). The population size was set to 10 million for sows and 0.5 million for wild boars, with a confidence level of 95%, a desired precision of ±5%, and an assumed seroprevalence of 10%. Based on these inputs, the minimum sample size required for both groups was calculated to be at least 139 animals. Accordingly, we collected 309 serum samples from sows (150 in 2019 and 159 in 2024) and 325 serum samples from wild boars (162 in 2019 and 163 in 2024; Table 1).

### 2.2. RT-PCR and Sequencing of the PSV Genome

RNA was extracted from fecal samples and culture supernatants, and subjected to reverse transcription polymerase chain reaction (RT-PCR) using primers specific for PSV in a previous study [32]. Additionally, fecal samples were screened using RT-PCR in previous studies to detect multiple potential pathogens, including Porcine Reproductive and Respiratory Syndrome Virus (PRRSV), Porcine Circovirus Type 2 (PCV2), Porcine Epidemic Diarrhea Virus (PEDV), and Porcine Rotavirus (PoRV) to evaluate co-infections and overall pathogen prevalence [33,34]. For further genomic analysis, eight sets of primers were designed to amplify the PSV genome (Table 2). Total RNA was extracted from viral supernatants using an RNeasy Mini Kit (Qiagen, Cat. No. 74104, Germantown, MD, USA) and then used for cDNA synthesis following the protocol provided in the HelixCript Kit (NanoHelix, Daejeon, Republic of Korea). PCR amplification of the PSV genome was performed using AccuPower ProFi Taq PCR Premix (Bioneer, Daejeon, Republic of Korea). The PCR conditions were as follows: initial denaturation at 95 °C for 5 min, followed by 35 cycles of 95 °C for 20 s (denaturation), 58 °C for 30 s (annealing), and 68 °C for 1 min (extension), with a final extension step at 68 °C for 5 min. The amplified PCR products were subjected to agarose gel electrophoresis, visualized using an ultraviolet image analyzer, and subsequently sequenced (Cosmogentech, Daejeon, South Korea). In addition, the AnyQvet PSV/PEV/PTV qRT-PCR kit (KOREAGENE, Chuncheon, Republic of Korea) was used to confirm the viral load of PSV in piglet feces. qRT-PCR conditions were as follows: 50 °C for 15 min (1 cycle), 95 °C for 5 min (1 cycle), 95 °C for 10 s/60 °C for 40 s (40 cycles).

### 2.3. Cell Lines and Virus Isolation

Swine testicular (ST) cells were cultured at 37 °C/5% CO_2_ in Dulbecco’s Modified Eagle’s Medium (DMEM; Gibco, Waltham, MA, USA) supplemented with 10% fetal bovine serum, 1% antibiotic-antimycotic (Anti-Anti; Gibco, Waltham, MA, USA), and 1% non-essential amino acids (NEAA) solution (Gibco, Waltham, MA, USA). For virus isolation, only PSV-positive fecal samples were processed. Briefly, fecal samples confirmed to be PSV positive were suspended in phosphate-buffered saline (PBS) to prepare a 10% (*w*/*v*) suspension, clarified by centrifugation, and filtered through a 0.22 μm membrane filter (Merck Millipore, Burlington, MA, USA). The filtered supernatant (1 mL) was then mixed at a 1:1 ratio with DMEM supplemented with 3 μg/mL of trypsin (Gibco, Waltham, MA, USA), 1% Anti-anti, and 1% NEAA before being inoculated onto confluent ST cells. The inoculated cells were incubated for 1 h at 37 °C/5% CO_2_, after which the inoculum was removed and the cells were washed twice with PBS before being maintained in serum-free DMEM. The cultures were monitored daily for cytopathic effects (CPEs). At 3 days post-infection (dpi), the cells underwent three freeze−thaw cycles prior to passage onto fresh ST cell monolayers (seven consecutive passages). The RT-PCR of cell lysates was at each passage to confirm the presence of the virus.

### 2.4. Serum Neutralization

Serum samples collected from sows and wild boars were heat-inactivated at 56 °C for 30 min and stored at −20 °C until use. To detect PSV-specific neutralizing antibodies, the serum samples were serially diluted two-fold in medium containing 3 μg/mL of trypsin (Gibco, Waltham, MA, USA). Briefly, 50 μL of the serially diluted serum sample was mixed 1:1 with 50 μL of the PSV/Goryeong/KR-2019 strain (200 TCID_50_/mL) isolated in this study and then incubated at 37 °C for 1 h. The virus/serum mixture was then inoculated onto confluent ST cell monolayers in 96-well plates and incubated for an additional 1 h at 37 °C. After incubation, the inoculum was removed and replaced with 100 μL of serum-free DMEM containing 3 μg/mL of trypsin. The cells were then cultured at 37 °C/5% CO_2_ for 3 days. At 3 dpi, CPEs were examined under a microscope to determine the presence of PSV-specific neutralizing antibodies. The PSV positive antibody by the neutralization test was set to ≥4-fold for clear judgment.

### 2.5. Virus Growth Kinetics

To evaluate the growth kinetics of isolated PSV strains, ST cells were infected at a multiplicity of infection (MOI) of 0.01. Cell supernatants were collected at 1, 6, 12, 24, 36, 48, 60, and 72 h post-inoculation (hpi). Viral titers were determined by 10-fold serial dilution in ST cells seeded in 96-well plates, and calculated as the 50% tissue culture infectious dose (TCID_50_) per mL using the Reed−Muench method [35]. Data from three replicate experiments were used for growth curve analysis.

### 2.6. Phylogenetic and Recombination Analysis

The complete ORF and VP1 gene sequences of PSVs examined in this study were analyzed alongside those of 75 and 72, respectively, global reference strains obtained from the NCBI GenBank database. Multiple sequence alignments were performed using BioEdit software (version 7.0.5.3). Phylogenetic analysis of the nucleotide sequences was conducted using the maximum-likelihood (ML) method, with the Tamura–Nei model and bootstrap analysis (*n* = 1000) in MEGA software (version 6.06) under default parameters. To investigate for potential recombination between the PSV/Goryeong/KR-2019 strain and 74 PSV reference strains, RDP (version 4.1.0) and Simplot (version 3.5.1) software were used.

### 2.7. Experimental Infection of Piglets and Fattening Pigs

To evaluate induction of diarrhea in suckling piglets by the isolated PSV/Goryeong/KR-2019 virus, six 5-day-old piglets (free of PSV antibodies and antigen) were divided into two groups of three. During the animal experiment, 5-day-old piglets were separated from their sows and fed a commercial milk substitute (Purina Co., Seongnam, South Korea) four times per day, with free access to water. One group was orally inoculated with 5 mL of PSV/Goryeong/KR-2019 (10^4^ TCID_50_/mL) virus, and the other was inoculated with 5 mL of DMEM as a negative control. Body weight, diarrhea, vomiting, and appetite were monitored for 4 days. In addition, fecal samples were collected before and 1–4 days post-virus (PSV/Goryeong/KR-2019) inoculation, and the presence of PSV infection was analyzed by qRT-PCR. Pigs infected with PSV/Goryeong/KR-2019 were euthanized at 4 dpi, and tissue samples were fixed in 4% paraformaldehyde for histopathological observation. In addition, five 130-day-old fattening pigs (free of PSV antibodies and antigen) were used to determine whether neurological clinical signs were induced by the isolated PSV/Goryeong/KR-2019 virus. Three 130-day-old pigs were inoculated intramuscularly with 5 mL (10^4^ TCID_50_/mL) of PSV/Goryeong/KR-2019 virus, and the other two served as negative controls. All pigs were observed for 3 weeks before being euthanized and examined for tissue lesions.

### 2.8. Statistical Analysis

All statistical analyses were performed using GraphPad Prism (version 8.0, GraphPad Software, San Diego, CA, USA). Data were expressed as mean ± standard deviation (SD). Pairwise comparisons between each experimental group and the MOCK group were conducted using Student’s *t*-test. Differences were considered statistically significant at *p* < 0.05.

## 3. Results

### 3.1. Seroprevalence of PSV

**Figure 1:**
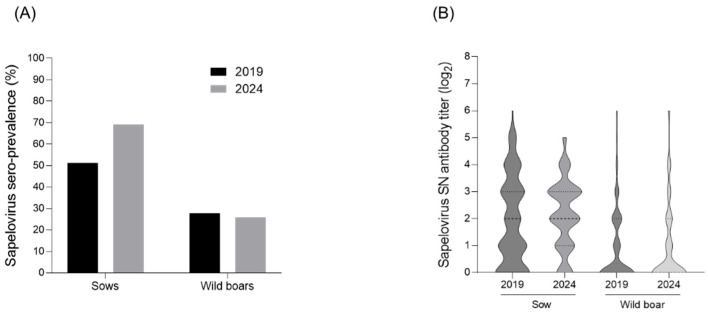
Changes in seroprevalence and antibody levels against PSV in wild boars and sows. (**A**) Seroprevalence and (**B**) distribution of neutralizing antibodies in sows and wild boars between 2019 and 2024.

Neutralization tests were conducted to investigate the serological changes in PSV levels in sows and wild boars. In 2019, a total of 150 sow serum samples were collected from 54 farms nationwide, with 51.3% (77/150) showing titers of ≥4-fold (Figure 1A,B). In 2024, 159 sow serum samples were collected from 53 farms, and 69.8% (110/159) exhibited titers of ≥4-fold, indicating an increase compared with 2019 (Figure 1A,B). For wild boars, 162 serum samples were collected in 2019, with 27.8% (45/162) showing titers of ≥4-fold (Figure 1A,B). In 2024, a total of 163 wild boar serum samples were analyzed, and 25.7% (42/163) had titers of ≥4-fold, a slight decrease compared with 2019 (Figure 1A,B).

### 3.2. Single or Co-Infection Analysis of PSV

Fifty-seven samples were analyzed to investigate the prevalence and co-infection rates of PSV among pigs. PSV was identified as a single infection in 21.1% (12/57) of samples (Table 3). Co-infections with other viruses were also detected; PSV and PRRSV co-infection was observed in 5.3% (3/57) of samples and PSV and PCV2 or PEDV co-infection was detected in 1.8% (1/57) (Table 3). Additionally, co-infection with PSV and Rotavirus was found in 5.3% (3/57) of samples, and triple infections involving PSV, PRRSV, and PCV2 were present in 7.0% (4/57) of samples (Table 3). PRRSV was detected as a single infection in 12.3% (7/57) of samples (Table 3). Other viruses, including PCV2, PEDV, and Rotavirus, were identified as single infections in 1.8% (1/57) to 5.3% (3/57) of samples (Table 3). Co-infections not involving PSV were also observed, with PRRSV and PCV2 co-infections detected in 3.5% (2/57) of samples, and PCV2 and PEDV co-infections found in 1.8% (1/57) (Table 3).

### 3.3. Isolation of PSV

**Figure 2:**
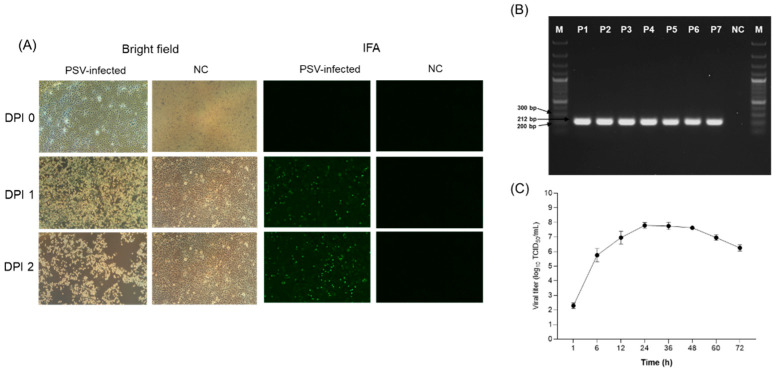
Isolation and identification of PSV. (**A**) Cytopathic effects (CPEs) and immunofluorescence assay (IFA) results of ST cells inoculated with PSV-positive samples. (**B**) RT-PCR of viral RNA in cell culture supernatants obtained from serially passaged PSV-positive samples. (**C**) Growth kinetics of PSV in ST cells.

To isolate the PSV virus from 12 PSV single-infection positive samples, the samples were inoculated onto ST cells; however, after seven blind passages, only one of the 12 samples showed CPE, characterized by rounding, shrinking, and detachment of the cells (Figure 2A). RT-PCR was performed to confirm isolation of the virus strain, and viral RNA was detected in the supernatant of serial passages of each isolate from only one sample. The virus that did cause CPE was detected by RT-PCR, with a band at 212 bp (Figure 2B). Analysis of virus growth curves revealed rapid replication, reaching a maximum titer of 10^7^ TCID_50_/mL at 24 h, indicating that the PSV replication cycle was completed within 24 h (Figure 2C). The isolated PSV strain was named PSV/Goryeong/KR-2019.

### 3.4. Phylogenetic Analysis of PSV

**Figure 3:**
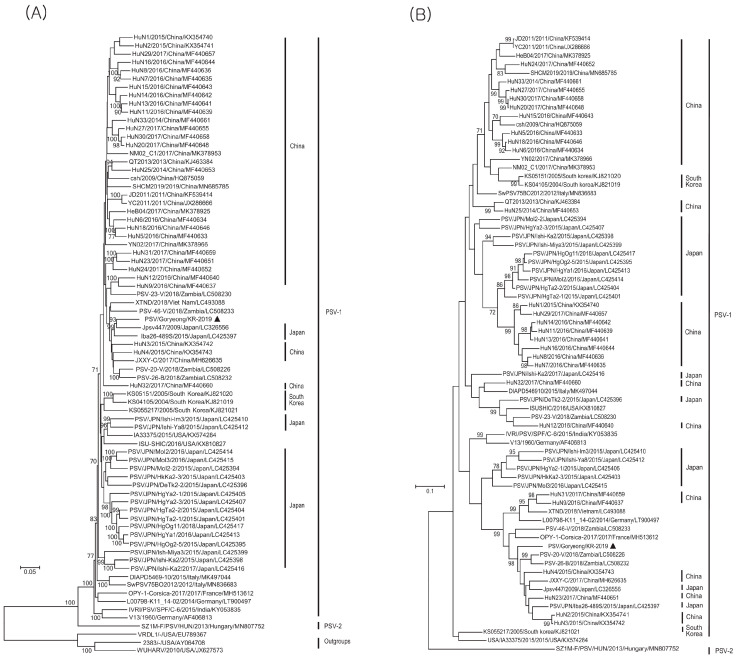
Phylogenetic trees of PSV ORF and VP1. (**A**) ML tree based on (6969–7266 nucleotide) 75 complete open reading frame (ORF) sequences including those of the PSV/Goryeong/KR-2019 strain and 74 reference strains, and (**B**) Phylogenetic analysis based on (858–894 nucleotide) 72 VP1 gene sequences including those of the PSV/Goryeong/KR-2019 strain and 71 reference strains. The trees were constructed using the maximum-likelihood method (with the Tamura–Nei model) and 1000 bootstrap replications.

The phylogenetic tree based on the polyprotein gene sequences revealed the presence of two major PSV genotypes: PSV1 and PSV2. PSV1 comprised the majority of the identified PSV strains, whereas PSV2 included only strains isolated from Hungary. The isolated PSV/Goryeong/KR-2019 strain was related most closely to the Jpsv447 strain isolated in Japan in 2009 (also the PSV1 genotype) (Figure 3A). The phylogenetic tree based on VP1 gene sequence confirmed that the PSV/Goryeong/KR-2019 strain belonged to the same PSV1 clade as the three PSV isolates isolated in Korea in 2004 and 2005. It also revealed high genetic homology and a close relationship with PSV-20V isolated in Zambia in 2018 (Figure 3B).

### 3.5. Recombination Analyses

**Figure 4:**
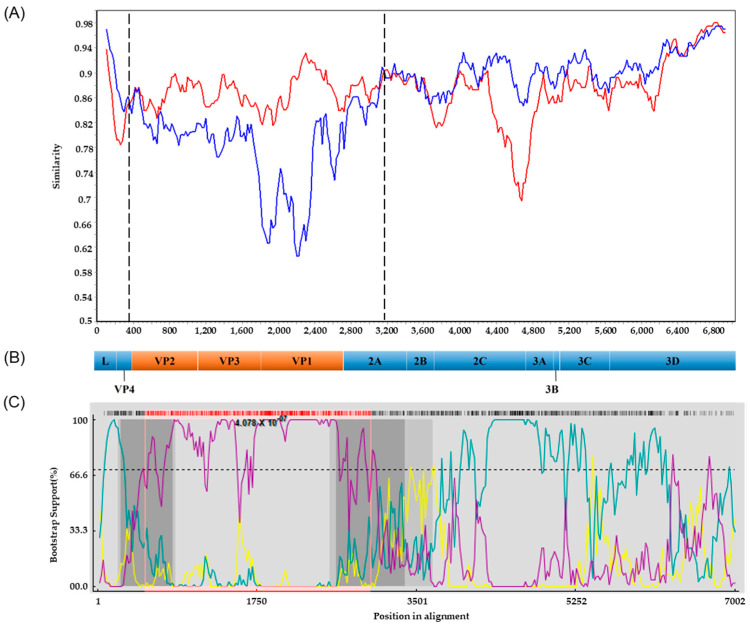
Recombination analysis of the PSV/Goryeong/KR-2019 strain. (**A**) Similarity plot generated using SimPlot (version 3.5.1) software (window size: 200 bp; step size: 20 bp; Kimura 2-parameter model). The similarity between the Jpsv447 (Japan, minor parent) strain (red line) and the KS05151 (Korea, major parent) strain (blue line) was compared based on the PSV/Goryeong/KR-2019 strain. (**B**) Schematic representation of the PSV genome, showing the coding regions for structural (VP4–VP1) and nonstructural (2A–3D) proteins. (**C**) BootScan analysis performed using the RDP4 program. The yellow line represents the recombinant strain PSV/Goryeong/KR-2019, the teal line indicates the minor parent strain KS05151 (South Korea), and the purple line indicates the minor parent strain Jpsv447 (Japan).

Potential recombinations involving the PSV/Goryeong/KR-2019 strain were analyzed using the standard similarity plot analysis. The VP2–VP3–VP1 region and the 5′ half of the 2A gene (369–3168 nt) of PSV/Goryeong/KR-2019 exhibited high nucleotide sequence similarity with the Japanese strain Jpsv447. By contrast, the L-V4 region, the 3′ half of the 2A gene, the 2B–2C region, and the P3 region showed high similarity to the Korean strain KS05151 (Figure 4A,B). To further characterize the recombination events, we performed breakpoint analysis using seven different methods (RDP, GENECONV, BootScan, MaxChi, Chimaera, SiScan, and PhylPro) implemented in the RDP4 (version 4.1.0) software package. Putative recombination breakpoints were identified at 263–820 nt and 2641–3380 nt, which are indicated as dark gray regions in the RDP4 analysis (Figure 4B,C). Additionally, BootScan analysis detected recombination breakpoints at 524–3000 nt, represented by the pink lines (Figure 4B,C). These recombination breakpoints were located within the VP2 and 2A regions, and with statistical significance (*p* = 4.078 × 10^−7^).

### 3.6. Clinical Signs and Histopathological Examination

**Figure 5:**
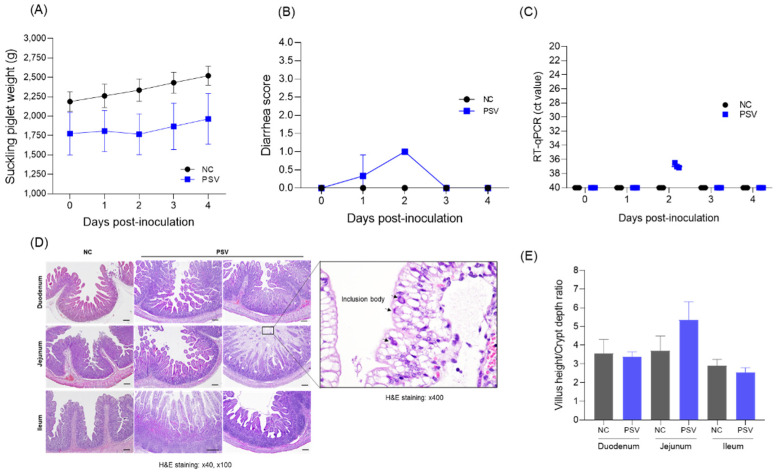
Clinical signs and necropsy results for suckling piglets. (**A**) Body weight changes. (**B**) Diarrhea scores. (**C**) PSV RNA copy number in feces. (**D**) Villus height/crypt depth ratio. (**E**) Histopathological images. The PSV and control groups are marked as blue and black, respectively. Tissues were stained with hematoxylin and eosin (H&E). Scale bar = 100 μm.

Post-mortem examination of suckling pigs revealed that the body weight of pigs in the PSV-infected group increased from 1773 ± 477 g to 1960 ± 573 g, whereas that of pigs in the negative control group increased from 2186 ± 220 g to 2520 ± 210 g (Figure 5A). In terms of diarrhea scores, the PSV-infected group showed mild diarrhea only on Day 1 (0.3 ± 0.5) and Day 2 (1.0) (Figure 5B). The presence of PSV RNA was assessed in both the control and PSV-inoculated groups using real-time PCR analysis. In the control group, no amplification signals were detected in any sample, indicating the absence of PSV RNA. By contrast, three piglets in the PSV-inoculated group exhibited Ct values of 37.15, 36.45, and 37.00 at 2 dpi, suggesting transient viral replication. No amplification signals were detected from 3 dpi (Figure 5C). Histological analysis revealed that the difference in the duodenum villus length and crypt depth (VH:CD) between the PSV-infected group and the negative control group was 3.38 ± 0.23 and 3.54 ± 0.66, respectively, and that in the ileum was 2.54 ± 0.22 and 2.91 ± 0.28, respectively, showing no significant difference. There was a slight difference in the jejunum of the PSV-infected group (5.35 ± 0.9) compared with the control group (3.69 ± 0.67), but the difference was again not significant (Figure 5E). No severe epithelial detachment or necrosis was observed in the small intestine of the PSV-infected group, but mild structural changes such as ballooning degeneration and inclusions in the jejunum were observed in some cases (Figure 5D).

**Figure 6:**
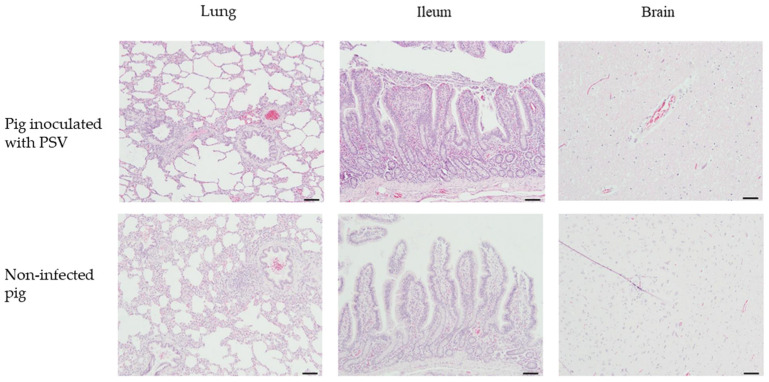
Histopathological images of lung, ileum, and brain tissues from PSV-infected and control fattening pigs. Tissues were stained with hematoxylin and eosin (H&E). Scale bar = 100 μm.

Histopathological examination of fattening pigs revealed mild inflammatory changes in the lungs and brain, with no significant differences between the PSV-infected and control groups (Figure 6). In the lung tissues, mild to minimal perivascular and peribronchiolar inflammatory cell infiltration was observed in all PSV-infected pigs, but similar findings were also noted in the control group (Figure 6). No notable lesions were observed in the ileum in either group (Figure 6). In the brain, minimal perivascular inflammatory cell infiltration was detected in some PSV-infected pigs, and a similar pattern of focal gliosis was occasionally observed in the control group (Figure 6).

In addition, three 5-day-old piglets inoculated with the isolated PSV/Goryeong/KR-2019 virus showed no antibodies to PSV at autopsy, and three 130-day-old fattening pigs formed approximately 7.3-fold antibodies (2-fold, 4-fold, and 16-fold).

## 4. Discussion

A recent Chinese study reported that nursery pigs had higher PSV viral loads, suggesting that maternal antibodies were decreasing and thus becoming the primary source of infection [18]. Although no positive samples were detected in suckling pigs, the prevalence of PSV in nursery pigs was high, suggesting that maternal antibodies present in suckling pigs may provide temporary immunity, and that maternal antibodies decrease with age, thereby increasing susceptibility to PSV infection [18]. In this study, we found that PSV antibody detection rates in sows increased by 18.5% between 2019 (51.3%) and 2024 (69.8%). Additional research will be conducted to investigate whether the recent PSV-antibody positivity in approximately 70% of pig farms is due to an increase in PSV on farms, to an external introduction of PSV, or to a combination of PSV and other diseases. In addition, although the PSV antibody rate in wild boars decreased slightly over 5 years (by 2.1%; from 27.8% in 2019 to 25.7% in 2024), we believe that this is because we tested wild boars of all ages; therefore, the rate will be lower than that in domestic sows. However, the presence of PSV antibodies in approximately 25% of wild boars can be considered evidence of ongoing circulating infections among them.

Frequent co-infection with PSV and other enteric pathogens complicates the identification of its specific role in clinical disease, often leading to its underestimation in diagnostic investigations and disease management strategies [25,27]. In our study, both single infections and mixed infections with other pathogens were investigated. PSV was identified as a single infection in 21.1% of the pig samples analyzed; however, high rates of co-infection or triple infection with various porcine viruses (i.e., PRRSV, PCV2, PEDV, and Rotavirus) were also observed. Although a PSV single infection in these pig farms may not have resulted in obvious clinical symptoms, it is possible to infer that co-infection with other pathogens may increase the disease severity in pigs [9]. Recent large-scale epidemiological surveys in China further support these findings [36]. In Henan Province, PSV was detected in 102 (22.17%) out of 460 clinical samples, among which 15.7% (16/102) were co-infected with PDCoV, 10.8% (11/102) with PEDV, and 5.9% (6/102) with TGEV [36]. Another study reported that 75 (40.54%) out of 185 samples were positive for PSV alone, and 32 (17.30%) for PSV/PEDV co-infection [9]. Notably, in diarrheic samples, the positive rates for PSV alone (44.93%, 62/138), PEDV alone (46.38%, 64/138), and PSV/PEDV co-infection (20.29%, 28/138) were significantly higher than those of non-diarrheic samples [9]. Moreover, PRRSV can damage the host immune system, increasing susceptibility to secondary infections [37], which may provide a favorable environment for PSV replication and long-term viral shedding. Similarly, PCV2, which is known to have immunomodulatory effects, is associated with increased viral load and long-term persistence of co-infected pathogens [38].

Phylogenetic analysis based on the complete polyprotein gene sequences clearly identified two major PSV genotypes, PSV1 and PSV2, a finding consistent with previous reports and indicated that PSV2 strains are restricted to Hungary [14,15,16,17,18]. The PSV/Goryeong/KR-2019 strain was classified within the PSV1 genotype, and was most closely related to the Jpsv447 strain isolated in Japan in 2009. This finding may reflect the close epidemiological relationship among strains circulating in neighboring countries. However, phylogenetic analysis of the VP1 gene revealed a different pattern, i.e., that PSV/Goryeong/KR-2019 is more closely related to the Zambian strain PSV-20V. Genetic variation and recombination are the central factors driving the evolutionary dynamics of picornaviruses, often resulting in inconsistent phylogenetic relationships across different genomic regions [39,40,41].

Recombination analysis using SimPlot and BootScan revealed that PSV/Goryeong/KR-2019 is a recombinant virus. Similarity plot analysis showed that the structural protein genes (VP2, VP3, and VP1) and the 5′ half of the nonstructural 2A gene are highly similar to the Japanese strain Jpsv447, while the L-V4 region, the 3′ half of the 2A gene, and the P2 (2A–3′, 2B, 2C) and P3 (3A, 3B, 3C, 3D) regions show higher similarity to the Korean strain KS05151. The recombination signals were detected at nucleotide positions 524 and 3000, and BootScan analysis further confirmed the presence of recombination breakpoints within the VP2 and 2A regions, consistent with the similarity plot analysis. Recombination is known to be a key mechanism driving the evolution of RNA viruses, and frequent recombination has been reported in viruses belonging to the Picornaviridae family, such as PSV, human enterovirus (EV), foot-and-mouth disease virus (FMDV), and EV-G [7,42,43,44]. In particular, the regions where recombination mainly occurs in the PSV genome have been suggested to be upstream of the 2B gene, the 3′ end of the P1 region, and the regions adjacent to the L and 2A regions [7,23,45]. However, a new recombination event was recently reported in China in the VP2 gene region [15]. In this study, recombination breakpoints were identified in the VP2 and 2A regions, which may support the possibility that, in addition to recombination at previously known recombination hotspots, recombination may occur more widely in other regions of the PSV genome. Recombination repeatedly observed near the VP1 and 2A gene boundaries may be the result of strong selection pressure by the host immune system to promote diversity in the surface antigen VP1 protein, which has also been suggested to be the reason for the frequent occurrence of genetic mutations, such as deletions and insertions as well as recombination at that site [14].

The biggest problem with PSV infection is that it is often asymptomatic, making it difficult to detect, and it is difficult to predict what role it will play and how much damage it will cause when pigs are simultaneously infected with PSV and other pathogens. In general, pig diarrhea viruses such as PSV are mainly transmitted through the fecal-oral route, but indirect transmission through contaminated transport trailers, workers, and feed is also frequent [46]. In particular, in farms with weak biosecurity, the risk of spread is very high when the swine diarrhea virus is introduced, resulting in significant economic losses; therefore, strict disinfection and enhanced farm biosecurity are essential [46].

Clinically, and similar to porcine circovirus type 2 (PCV2), PSV has a high infection rate in both domestic and wild pig populations [47]; however, most pigs infected with PSV do not show clinical signs [22,29,47,48], suggesting that this virus is consistently low-pathogenic. In the suckling pigs inoculated with the PSV/Goryeong/KR-2019 strain in this study, clinical signs were mild, body weight differences were minimal when compared with negative controls, and levels of virus shedding were low. Histopathological examination also revealed no significant differences between the infected and control groups. In addition, mild inflammatory changes were observed in the lungs and brain of growing pigs inoculated with the PSV/Goryeong/KR-2019 strain, but there was no statistically significant difference between the PSV-infected and control groups. Previous studies have reported that PSV causes diarrhea in piglets [9,22,49] and neurological symptoms in fattening pigs [10,11,13], but the results of this study suggest that the PSV/Goryeong/KR-2019 strain has low pathogenicity in suckling and growing pigs. In addition, in the PSV/Goryeong/KR-2019 strain inoculation experiment, there was no change in antibody titer in piglets, and fattening pigs showed low antibody titers; therefore, no clear seroconversion pattern was confirmed.

These low-pathogenic strains may not cause severe clinical disease when infected, but may circulate in the pig population, causing widespread asymptomatic infections. Furthermore, these asymptomatic infections may serve as reservoirs for virus transmission and may be more likely to exacerbate disease severity when the pigs are co-infected with PSV/Goryeong/KR-2019 and other pathogens (e.g., PRRSV, PCV2, PEDV). Therefore, continuous monitoring of PSV prevalence and pathogenicity and further studies on the impact of co-infection with other pathogens are essential for a more comprehensive understanding of the role of PSV in pig health and disease epidemiology.

Based on these results, we believe that the PSVs prevalent in South Korea are likely to be mainly low-pathogenic, although further research is needed to discover the role of PSV in promoting pathogenicity when mixed with other viruses.

## 5. Conclusions

PSV antibody seroprevalence in wild boars in South Korea decreased slightly from 2019 to 2024, but increased significantly in domestic sows, indicating active circulation in commercial pigs. Phylogenetic analysis identified all isolates as the PSV1 genotype, and animals inoculated with the PSV/Goryeong/KR-2019 strain had mild clinical signs, suggesting low pathogenicity. Although PSVs in South Korea are mainly low-pathogenic, continuous surveillance and genetic monitoring are essential for effective pig health management, particularly considering the possibility of co-infection with immunosuppressive viruses.

## Figures and Tables

**Table 1:** Serum samples background.

Province	2019		2024	
	Sows	Wild Boars	Sows	Wild Boars
Gangwon	10	12	12	12
Geonggi	30	30	27	30
Gyeongnam	25	27	27	28
Gyeongbuk	5	6	6	6
Jeonnam	5	6	6	6
Jeonbuk	20	21	21	21
Chungnam	35	36	36	36
Chungbuk	10	12	12	12
Jeju	10	12	12	12
Total	150	162	159	163

**Table 2:** Primers used to amplify the PSV genome.

Primer Name	Oligonucleotide Sequence (5′-3′)	Primer Position	Product Size (bp)
PSV-1F	GGACTTGGACCTCTGGCAA	1–19	1188 bp
PSV-1R	GCTGACCTGAGTTGGGGTTT	1168–1187	
PSV-2F	GACAGCCTGCAATGGACAAA	972–991	1163 bp
PSV-2R	TGCACTGACAGTAACCACCA	2115–2134	
PSV-3F	TCAGATGGCCACCGGTAAAT	1850–1869	1225 bp
PSV-3R	GGCCTAATTGTTCTGCAGGG	3054–3074	
PSV-4F	CGGAATGGTGCTTCTTATGGT	2821–2842	1261 bp
PSV-4R	TAAGCCACTCAGAAGGTCCC	4062–4082	
PSV-5F	GAATGCACGACTGGGTTCAA	3728–3748	1212 bp
PSV-5R	TTGCTTTGCCACAAACCAGT	4920–4940	
PSV-6F	ACAACCACCTACATTCCACCT	4660–4681	1209 bp
PSV-6R	ACCTCCACACTGTCCCATTT	5849–5869	
PSV-7F	TTTCACTGGACTGGGCATCT	5496–5516	1200 bp
PSV-7R	GGTGACAGACTAGCATCCCA	6676–6696	
PSV-8F	TGAGCTGAGACCCAAAGAGA	6444–6464	907 bp
PSV-8R	ATCCAACCAAGACCTACGCA	7331–7351	

**Table 3:** Non-infection, single-infection, and co-infection rates in pigs.

Virus	No. of Positive Samples /No. of Test Samples	Positive Rate (%)
PSV	12/57	21.1
PSV + PRRSV	3/57	5.3
PSV + PCV2	1/57	1.8
PSV + PEDV	1/57	1.8
PSV + Rota	3/57	5.3
PSV + PRRSV + PCV2	4/57	7.0
PRRSV	7/57	12.3
PCV2	1/57	1.8
PEDV	1/57	1.8
Rota	3/57	5.3
PRRSV + PCV2	2/57	3.5
PCV2 + PED	1/57	1.8

## Data Availability

Data are available from the corresponding author upon reasonable request.
